# Effect of doping on single-walled carbon nanotubes network of different metallicity

**DOI:** 10.1186/1556-276X-7-548

**Published:** 2012-10-03

**Authors:** Ju Nie Tey, Xinning Ho, Jun Wei

**Affiliations:** 1 Joining Technology Group, Singapore Institute of Manufacturing Technology, 71 Nanyang Drive, Singapore, 639798, Singapore

## Abstract

Effects of doping on single-walled carbon nanotubes (SWNT) networks with different metallicity are reported through the study of sheet resistance changes upon annealing and acid treatment. SWNT film with high metallic tube content is found to have relatively good chemical stability against post treatments, as demonstrated from its stable film performance in ambient after annealing, and merely 15% reduction in sheet resistance upon sulfuric acid treatment. Conversely, film stability of SWNT film with low metallic content which comprises largely of semiconducting SWNT varies with days in ambient, and its sheet resistance changes drastically after treated with acid, indicating the extreme sensitivity of semiconducting SWNT to surrounding environment. The results suggest that annealing removes unintentional oxygen doping from the ambient and shifts the Fermi level towards the intrinsic Fermi level. Acid treatment, on the other hand, introduces physisorbed and chemisorbed oxygen and shifts the Fermi level away from the intrinsic level and increases the hole doping.

## Background

Carbon nanotube (CNT) is an interesting nanomaterial. Ever since its discovery, single-walled carbon nanotube (SWNT) has been exhaustively studied with all types of characterization tools to understand its unique electrical, mechanical, and thermal properties [1-3]. Among all potential applications, the use of carbon nanotube for flexible transparent conducting film has shown to be a promising area [4-6]. The film conductivity of SWNT thin film arises from the carrier transport along the cylindrical sidewall and the carrier hopping from one tube to another: the higher the tube density, the better the conductivity, which can be understood in the framework of the percolation theory [7]. Intrinsically, single nanotube possesses supremely high electrical conductivity of approximately 10^6^ S/cm at room temperature [8], a value better than the conductivity of metals such as copper at room temperature. However, the interaction between numerous nanotubes of different properties in 2-D or 3-D networks complicates and alters the transport behavior. The tunneling barrier at the junction of two metallic-SWNTs contact and the junction of two semiconducting SWNTs contact, as well as the Schottky barrier between a metallic SWNT (M-SWNT) and semiconducting SWNT (S-SWNT) [9], results in that the random CNT network conducting films being unable to meet the film performance as expected theoretically.

CNT has been shown to be sensitive to chemical doping [10,11]. For film conductivity improvement, acid treatments have been proven effective. Studies of CNT films treated with nitric acid [12,13], thionyl chloride [12,13], sulfuric acid [14], etc. demonstrated increased electrical conductivity. It was understood that these redox dopants introduce hole doping into the CNT network and lower the Fermi level [15]. Very often, annealing step is performed after acid treatment, and hence the sheet resistivity change is a combined effect of both treatment processes. It is then interesting to look into the individual contribution of acid treatment and annealing to the conductivity of CNT network.

In this study, we investigated the impact of vacuum annealing and acid treatment on the SWNT network. In the process of evaluating the film performance, it was interesting to find that SWNT films of heterogeneous electronic types respond differently to the treatment process. We therefore included in this study the performance assessment of SWNT films with three different metallic tubes content, i.e., SWNT films prepared from 5%, 50%, and 90% M-SWNT (or 95%, 50%, and 10% S-SWNT).

## Methods

### CNT films preparation and post-treatment steps

**Figure 1:**
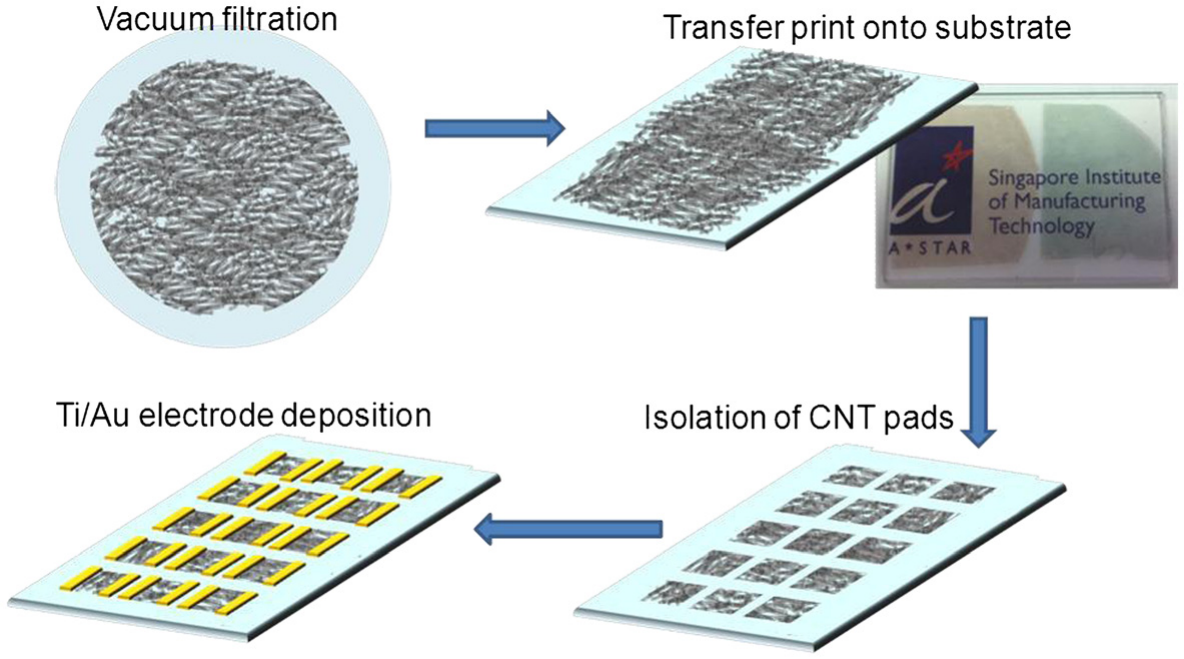
** llustration of SWNT films, pad isolation, and electrode deposition.** Illustration of SWNT films made by vacuum filtration, and pad isolation and electrode deposition for sheet resistance measurement.

95% S-SWNT and 90% M-SWNT were purchased commercially (IsoNanotubes-S and IsoNanotubes-M from NanoIntegris Inc, Menlo Park, CA, USA). A designated volume of solution was casted into thin films on alumina filter membranes (Whatman anodiscs, 47 mm, 0.1 μm; Whatman Ltd, Maidstone, Kent, UK) using the vacuum filtration method reported previously [16,17], followed by transfer printing onto glass substrates or polyethylene terephthalate (PET) substrates using polydimethylsiloxane (PDMS) stamp (Figure 1). 

The transferred SWNT films were rather thin and adhered weakly onto the glass substrate. The conventional method for sheet resistance (*R*_s_) measurement using four-point probe did not work well on the samples as the probe tips tend to scratch the surface easily and affect the measurement. In our sheet resistance measurement, we isolated the CNT film into islands and deposited Ti/Au electrodes at the two edges so that the final dimension of the measurable CNT area is 1 mm (*W*) × 1 mm (*L*). By making the *W* equals to *L* and *R*_s_ of the SWNT, films can then estimated from the following equation:

(1) $$R=\rho \frac{L}{Wt};R_{s}=R\times \frac{W}{L}=\frac{\rho }{t},where\;W=L$$

Where *ρ* is the resistivity; *W* and *L* refer to the width and length of the CNT area, respectively; *t* is the thickness of the film. One drawback of this measurement is that the contact resistance contributes to the measured resistance. Hence, the reported sheet resistance value might be higher than the actual sheet resistance. Nevertheless, since our objective is to understand the impact of post treatments and the changes are usually normalized to the original value, the trends and conclusions drawn from the experiment should not be affected.

After the electrode deposition, the SWNT films were subjected to thermal annealing at 200°C in vacuum, followed by acid treatment in 9 M H_2_SO_4_ for different durations. Sheet resistance of the each SWNT pad was recorded for as-prepared condition, after electrode deposition, after annealing step, and after acid treatment to observe the change in value after treatments. This is same for optical characterization.

### Electrical and optical characterization of CNT films

The sheet resistance of CNT was measured using Keithley 2600 sourcemeter (Keithley Instruments Inc., Cleveland, OH, USA). The recorded resistance value is equal to sheet resistance due to the patterning of the pad to ensure *W* = *L*. For optical characterization, UV-Vis spectrometer was used to estimate the film transparency and enable us to evaluate the changes in film properties by comparing the absorbance peaks before and after various treatment processes.

## Results and discussion

**Figure 2:**
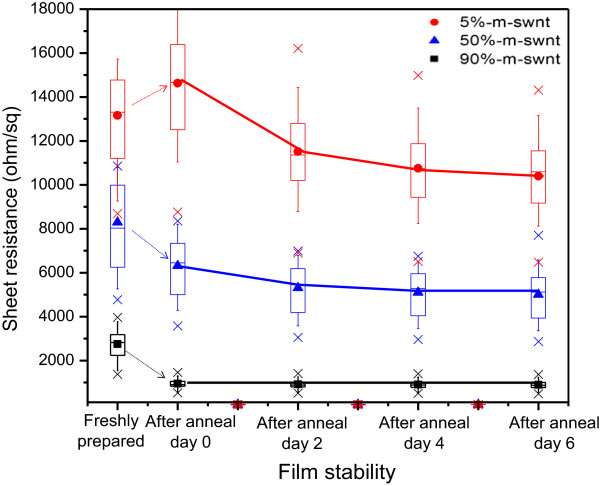
** Sheet resistance of SWNT films and its stability.** Sheet resistance of SWNT films before and after annealing, and its stability in ambient (up to 1 week) presented in box plot. The bottom and top of the box indicates the 25th and 75th percentile, and the band near the middle of the box refers to the 50th percentile. The mean sheet resistances are represented by the solid circle, triangle, and square symbols, with the maximum and minimum points indicated as the cross symbol in the graph.

The films' transparency was checked prior to SWNT pad isolation and electrode deposition, which fall in the range of 79% ± 2%, meeting the minimum requirement for transparent conducting films. The effect of annealing on SWNT films of different M-SWNT content was first examined, with results from the 30 measurements presented in Figure 2. It was found that M-SWNT gave positive response to annealing (Figure 2, red plot): the higher the metallic content, the better reduction in the sheet resistance. However, for SWNT film with almost no presence of metallic tubes, i.e., 95% pure S-SWNT, the sheet resistance worsened after annealing (Figure 2, black plot). The results showed that the behaviors of M-SWNT and S-SWNT are rather diverse after treatment. To further monitor the divergence in SWNT response, film stability check was carried out after annealing treatment. Repeat sheet resistance measurement up to 6 days indicated the good stability of high M-SWNT film. By having lesser metallic content in the network (increasing S-SWNT), the network became more sensitive towards ambient environment, with sheet resistance varied from daily measurement.

After annealing, the films were then immersed in 9 M sulfuric acid for 1 h. Sheet resistances were measured again after the films were taken out from the acid solution, rinsed with DI water, and blown-dried with N_2_ gas. Measurement results showed that the degree of sheet resistance changes after acid treatment is different from annealing: S-SWNT responded a >90% reduction in resistance after sulfuric acid treatment, while M-SWNT only gave approximately 15% reduction.

**Figure 3:**
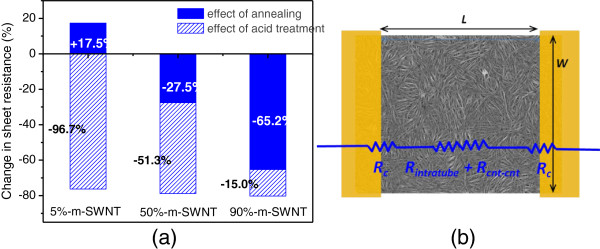
** Response to annealing and acid treatment and contribution of different resistance components to total sheet resistance.** (**a**) Response to annealing and acid treatment for SWNT films of different M-SWNT content. (**b**) Contribution of different resistance components to the total sheet resistance measured in this study.

The results from annealing and acid treatment are summarized and plotted in Table 1 and Figure 3a, which were compiled from more than 30 measurements per condition. The degree of response from SWNTs of different M-SWNT content can be clearly observed from the figure: SWNT films with lowest M-SWNT content (5% M-SWNT) gave deteriorated conductivity after annealing and improved drastically upon immersion in sulfuric acid; conversely, SWNT films with highest M-SWNT content (90% M-SWNT) exhibited tremendous conductivity improvement after annealing and little improvement after acid treatment.

**Table 1:** Summary of measured sheet resistances from different post treatments for SWNT films of varied metallic/semiconducting content

	**5% M-SWNT (95% S-SWNT) (%)**	**50% M-SWNT (50% S-SWNT) (%)**	**90% M-SWNT (10% S-SWNT) (%)**
After annealing	+17.5	−27.5	−65.2
After acid treatment	−96.64	−51.3	−15.0
Combined effect	−76.3(R_s_↓)	−78.8(R_s_↓)	−80.2(R_s_↓)

**Figure 4:**
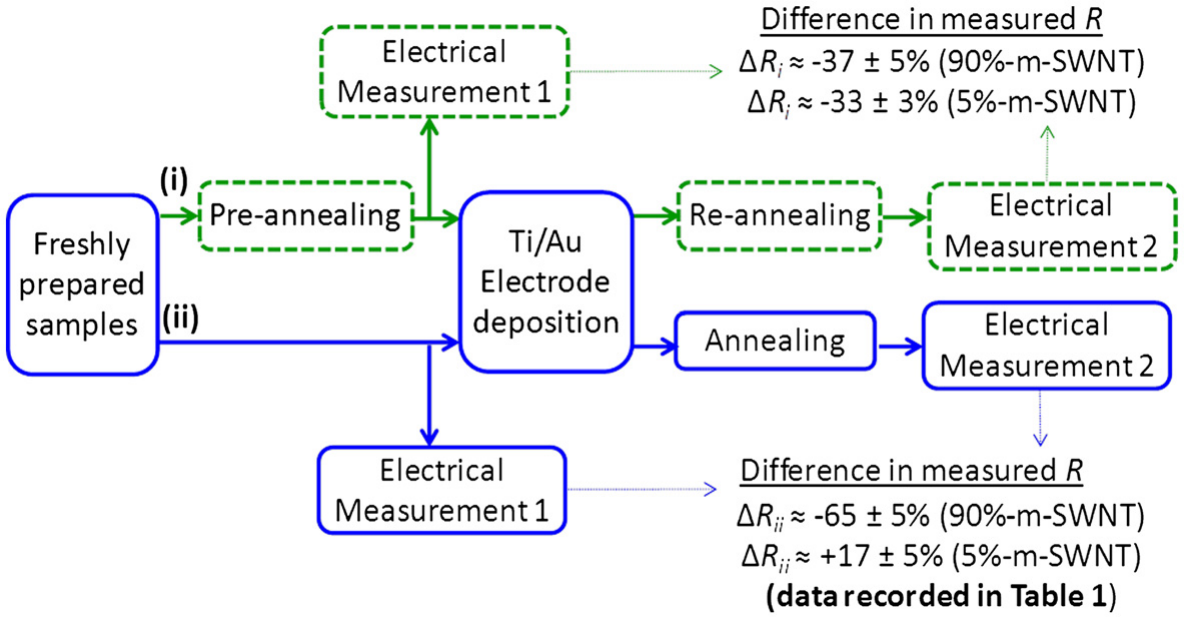
** Rough estimation for contact resistance (*R***_**c**_**) at the electrode-SWNTs interface.** The *ΔR*_ii_ (values reported in Table 1) is the result of *R*_c_, *R*_intratube_, and *R*_cnt-cnt_ changes upon annealing. With additional annealing step (case (i)) carried out before electrode deposition, lesser resistance reduction was observed (*ΔR*_i_ <*ΔR*_ii_). This resistance change (*ΔR*_i_) is deduced to be the *R*_c_ contribution from the metal electrode.

In the process of interpreting the experimental results, we did concern about the interference from contact resistance (*R*_c_) to the total changes of sheet resistance measured, as highlighted earlier in the experimental section. Based on the device structure used in our study (see Figure 1), the resistance value measured is the summation of contact resistances (*R*_c_), intratube resistances (*R*_intratube_), and tube-to-tube resistances (*R*_cnt*-*cnt_) of a SWNT network (Figure 3b), where *R*_c_ is due to the mismatch of the Fermi energy at the interface between metal electrodes and SWNTs; *R*_intratube_ depends on the existence of defects along the sidewall of a SWNT; *R*_cnt-cnt_ is the contact resistance between two SWNTs of different electronic properties, which causes tunneling barrier for the carrier at the intersection of two SWNTs. Of the three resistances, the latter two terms contribute to the true sheet resistance of the SWNT film. To estimate the effect of *R*_c_, we performed a qualitative measurement by preannealing a set of 5% and 90% M-SWNT films (200°C, 1 h in vacuum) prior to electrode deposition, followed by reannealing after electrode deposition (Figure 4, case (i)) and compared the measurement results to the standard sets where annealing was carried out only after electrode deposition (Figure 4, case (ii); results for these standard sets are tabulated in Table 1). In case (i), since preannealing was done on the SWNT network before electrode deposition, the resistance changes (*ΔR*_i_) upon re-annealing after electrode deposition are hence attributed to the *R*_c_ contribution from electrode-SWNT interface, assuming that SWNT network (*R*_intratube_ + *R*_cnt-cnt_) has been annealed and improved during the pre-annealing stage and remained unchanged upon re-annealing. This reduction is appreciably varied from *ΔR*_ii_, which implies that annealing does have an impact in the sheet resistance of the SWNT films. Depending on the electronic type of SWNT, M-SWNT experienced improved sheet resistance, while S-SWNT experienced deteriorated sheet resistance after annealing.

**Figure 5:**
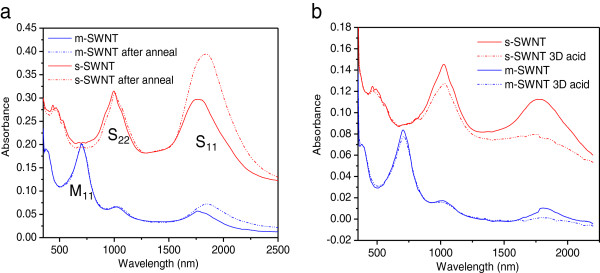
** UV-Vis spectra for SWNT films.** (**a**) Effect of annealing. (**b**) Effect of acid treatment.

To gain insights into the origins of the film performance's changes upon post treatments, UV-Vis spectroscopy measurement was collected for SWNT films before and after annealing and acid treatment. Figure 5 shows UV-Vis spectra for S-SWNT films (5% M-SWNT, red) and 90% M-SWNT (blue). The optical absorbance peaks indicate the electronic transitions between van Hove singularities (vHS) above and below the intrinsic Fermi level (E_Fi_) [18,19]. S_11_ and S_22_ are interband energy transitions from first and second vHS in S-SWNT, which can be seen from 95% S-SWNT spectra. M_11_ corresponds to the intraband transition in M-SWNT, which is the dominating peak in 90% M-SWNT (Nanointegris Technical Data Sheet). The small S_11_ and S_22_ peaks that were observed in 90% M-SWNT belong to the 10% S-SWNT mixture in the network. It is apparent from the UV-Vis measurement that S-SWNT varies considerably with post treatments, as one can see from the strong raise in S_11_ peak upon annealing and bleach upon acid treatment. Compare to S-SWNT, the M-SWNT exhibits smaller changes. 

**Figure 6:**
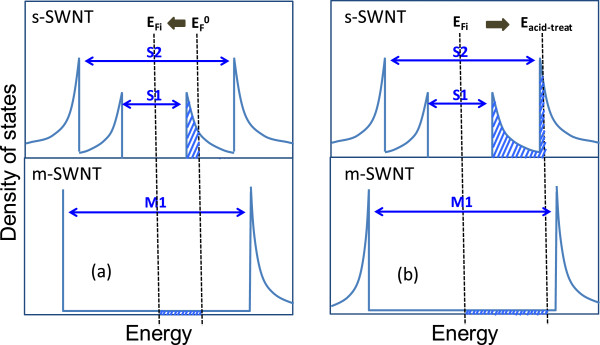
** Illustration of density of states for S-SWNT and M-SWNT with different dopings and the shift in Fermi level.** (**a**) Effect of annealing. (**b**) Effect of acid treatment.

It has been shown that oxygen and moisture absorbs readily on the surface of SWNT. The response from S-SWNT and M-SWNT to annealing and acid treatment can hence be attributed to the hole de-doping and doping effect which are contributed by oxygen or water molecules. The details could be better explained by a change in density of states (DOS) at the Fermi level due to the charge transfer between the SWNTs and absorbed molecules, as illustrated in Figure 6 (the DOS vs*.* energy diagram) [19]. vHS induce huge electronic DOS at the edges of valence and conduction bands, as was exemplified by the peaks shown in the figure, which represent the S11, S22, and M11 bandgaps. As-prepared SWNT network consists of unintentional doping from the ambient, which moves the Fermi level to near or slightly below the S_11_ hole level [20], and denoted as E_F_^0^ in Figure 6a. In the case of S-SWNT, vacuum annealing removes oxygen and moisture from the S-SWNT film, shifting the E_F_^0^ towards intrinsic Fermi level (E_Fi_). This shift in Fermi level is apparent from the reappearance of S_11_ peak in Figure 5a after annealing. The de-doping process reduces hole carrier concentration and hence results in the sheet resistance of S-SWNT being increased significantly. 

For the case of M-SWNT, since M-SWNT has constant DOS near the Fermi level, the Fermi level shift has little effect on the doping. Nevertheless, we still observed improved electrical conductivity in 90% M-SWNT. The improvement could be contributed from the drying of residual surfactant and film densification [21] after annealing, which leads to a better tube to tube contact (*R*_cnt-cnt_). In addition, annealing also leads to lower surface work function of M-SWNT. For Nanointegris M-SWNT with tube diameter range of 1.2 to 1.7 nm, the work functions are calculated to be 4.75 to 4.77 eV based on first principles calculations [22]. Annealing shifts the work function towards the intrinsic value, making it more compatible with Ti interface (4.33 eV), and facilitates the junction conductance.

The subsequent acid treatment in strong oxidizing sulfulric acid, on the other way, shifts the Fermi level away from the intrinsic level. The treatment has low impact on M-SWNT because of the constant DOS throughout the Fermi level shifting (−15% in *R*_s_) but was significant on S-SWNT. Acid treatment leads to O_2_ doping either through physisorption on the SWNT surface, or chemisorptions with hydroxyl (-OH) or carboxyl (-COOH) formation on the dangling bonds or defects [23]. Oxygen, with strong electronegativity, acts as electron acceptor and increases hole density in SWNTs. This is clearly illustrated in Figure 6b, which shows the shifting of Fermi level into the second vHS band of S-SWNT (*E*_acid-treat_), and is evidenced by the quench of S_11_ peak and reduced S_22_ peak from UV-Vis spectra (Figure 5b). Thus, carrier density increases and conductivity improves.

**Figure 7:**
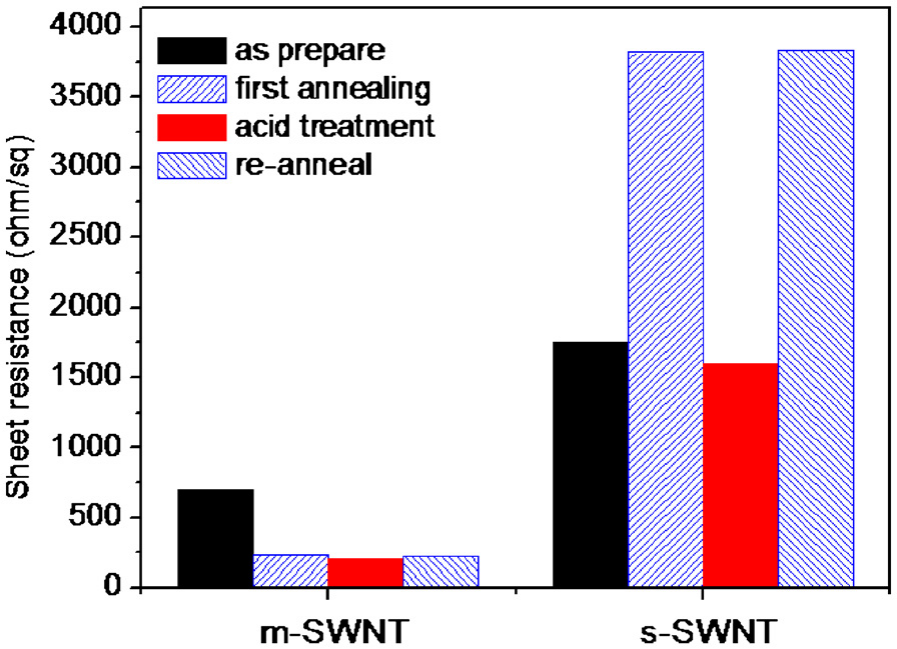
** Comparison of sheet resistance between 90% M-SWNT and 95% S-SWNT film at different treatment process.** The effect of acid treatment is shown to be completely reversible upon vacuum annealing.

Dopant hole density estimation performed by Blackburn et al. suggested that the drastic reduction of sheet resistance cannot be solely due to the increase in total carrier density as the improvement is far beyond the 1% carrier density increment at single-tube level [20]. It was thought that the O_2_ molecules absorbed at or near the tube-tube junctions create local electric fields and reduce the tunnel barriers, thus improving *R*_cnt-cnt_ and increase the degree of carrier delocalization. It is to be noted also that if the conductivity improvement is dependent on acid treatment condition, better result can be obtained with higher sulfuric acid concentration and longer immersion duration, and the process is completely reversible (Figure 7). 

## Conclusions

In summary, we evaluated the effects of annealing and acid treatment on SWNT films of different M-SWNT content. It was found that M-SWNT is more chemically stable than S-SWNT, as was shown from their response to acid treatment and doping, as well as the performance stability in ambient. Annealing removes absorbed O_2_ and water molecules from the SWNT network, shifts the Fermi level towards intrinsic Fermi level, and reduces hole carrier density. The impact is visible for S-SWNT from the considerable worsened sheet resistance after annealing. For M-SWNT, de-doping has not much effect on the carrier density. Reduction in sheet resistance is hence assumed to be from the better tube-to-tube contact (*R*_cnt-cnt_) and lowered surface work function of annealed M-SWNT, which leads to better carrier flow at the interface. Acid treatment, on the other hand, improves the conductivity through few means: (1) introduces O_2_ doping to increase hole density, (2) reduces tunneling barriers at tube-tube intersection (*R*_cnt-cnt_), and (3) increases the degree of carrier delocalization to facilitate charge hopping. The impact of acid treatment is very prominent in S-SWNT due to its higher chemical reactivity as compared to M-SWNT. Although the total improvement in S-SWNT is higher than M-SWNT, we found in our experiment that the conductivity of treated M-SWNT film is still superior. Nevertheless, the better chemical reactivity of S-SWNT allows for further potential improvement from doping treatment with other acids or strong oxidizers.

## Competing interests

The authors declare that they have no competing interests.

## Authors' contributions

JNT conceived of the study, designed and executed the experiment, and performed the statistical analysis. XH participated in sequence alignment and manuscript drafting. JW participated in the design of the study. All authors read and approved the final manuscript.

## Authors' information

JNT is a scientist of the Joining Technology Group in Singapore Institute of Manufacturing Technology. Her research interest is on carbon nanomaterials development, device fabrication, and characterization. Her recent research focuses on carbon nanotube-based organic electronics for biosensing and photovoltaic application. JW is a senior scientist and group manager of the Joining Technology Group in Singapore Institute of Manufacturing Technology. His research interests include carbon nanotubes, graphene, and other 1D and 2D nanomaterials used for the development and applications of devices and nanocomposites.
